# Effect of the US-Mexico border region in cardiovascular mortality: ecological time trend analysis of Mexican border and non-border municipalities from 1998 to 2012

**DOI:** 10.1186/s12889-017-4332-6

**Published:** 2017-05-06

**Authors:** Gabriel Anaya, Wael K Al-Delaimy

**Affiliations:** Department of Family Medicine and Public Health, Division of Global Health, University of California, 9500 Gilman Dr, La Jolla, San Diego, CA 92093 USA

**Keywords:** Cardiovascular Disease, Mexico, International Border, US-Mexico Border, Risk factors, Cardiovascular Mortality

## Abstract

**Background:**

An array of risk factors has been associated with cardiovascular diseases, and developing nations are becoming disproportionately affected by such diseases. Cardiovascular diseases have been reported to be highly prevalent in the Mexican population, but local mortality data is poor. The Mexican side of the US-Mexico border has a culture that is closely related to a developed nation and therefore may share the same risk factors of cardiovascular diseases. We wanted to explore if there was higher cardiovascular mortality in the border region of Mexico compared to the rest of the nation.

**Methods:**

We conducted a population based cross-sectional time series analysis to estimate the effects of education, insurance and municipal size in Mexican border (*n* = 38) and non-border municipalities (*n* = 2360) and its association with cardiovascular age-adjusted mortality rates between the years 1998–2012. We used a mixed effect linear model with random effect estimation and repeated measurements to compare the main outcome variable (mortality rate), the covariates (education, insurance and population size) and the geographic delimiter (border/non-border).

**Results:**

Mortality due to cardiovascular disease was consistently higher in the municipalities along the US-Mexico border, showing a difference of 78 · 5 (95% CI 58 · 7-98 · 3, *p* < 0 · 001) more cardiovascular deaths after adjusting for covariates. Larger municipal size and higher education levels showed a reduction in cardiovascular mortality of 12 · 6 (95% CI 11 · 4-13 · 8, *p* < 0 · 001) deaths and 8 · 6 (95% CI 5 · 5-11 · 8, *p* < 0 · 001) deaths respectively. Insurance coverage showed an increase in cardiovascular mortality of 3 · 6 (95% CI 3 · 1-4 · 0, *p* < 0 · 001) deaths per decile point increase. There was an increase in cardiovascular mortality of 0 · 3 (95% CI −0 · 001-0 · 6, *p* = 0 · 050) deaths per year increase in the non-border but a yearly reduction of 2 · 9 (95% CI 0 · 75-5.0, *p* = 0 · 008) deaths in the border over the time period of 1998–2012.

**Conclusion:**

We observed that the Mexican side of the US-Mexico border region is disproportionately affected by cardiovascular disease mortality as compared to the non-border region of Mexico. This was not explained by education, population density, or insurance coverage. Proximity to the US culture and related diet and habits can be explanations of the increasing mortality trend.

## Background

Chronic diseases have strongly impacted the health system of most nations and disproportionately affect developing nations [1, 2]. Cardiovascular diseases (CVD) are amongst the most common chronic diseases and have the highest mortality in the US, Mexico and the world. The US-Mexico border is one of the largest extending borders in the world with 1,954 miles in length and integrates 47 border crossings; including the San Ysidro-Tijuana border, the most transited border in the world. In both the US and Mexico, the border region is considered medically underserved [3], with a population that has pressing health and social conditions, higher uninsured rates [3, 4], high rates of migration [3], inequitable health conditions and high poverty rates [4]. Economic opportunity along Mexico’s border with the United States has been a driver of migration from across Mexico to the border region. Although improvements in health infrastructure and coverage in Mexico’s border reflect national trends, social and economic dynamics differ from the rest of the country [5].

The US-Mexico border region has received growing research interest with the development of multiple binational collaborations and efforts to better understand the health and risks in both sides of the border [6], but population research on chronic diseases is still limited. The economic and social implications of premature mortality owed to CVD require the identification and prevention of the elements that precipitate this rising mortality trend [2, 7]. Unfortunately, in many developing countries, risk factor data is limited and/or is descriptive of the country as a whole or that of a single State. This broad evaluation of risk varies greatly by regions and sometimes doesn’t reflect the rates in the community [5, 8]. In this research paper we will evaluate CVD mortality over time and compare US-Mexico border and non-border municipalities while incorporating available elements specific to those municipalities.

The aim of our manuscript is to better understand the health outcomes on the Mexican side of the U.S.-Mexico border in terms of cardiovascular mortality. We hypothesize that the Mexican border area along the U.S.-Mexico border suffers more from CVD mortality than the rest of Mexico. Quantifying these differences is a first step to gaining a better understanding of the social determinants of health along the US-Mexico border.

## Methods

In this study we adjusted population distribution at age of death and standardized CVD mortality rates in the US-Mexico border municipalities and non-border municipalities of Mexico to evaluate the effects of ecological factors such as education level, insurance rate and municipal size on CVD mortality. We defined our “Border Region” by including the *n* = 37–38 municipalities that share part of the US-Mexican border and our “Non-Border” with *n* = 2,270–2,360 municipalities that do not share part of the US-Mexico border. We selected the border municipalities because of the reported higher mortality [3] in the US-Mexico border region. Mortality was adjusted using Mexico’s National Population Council (CONAPO) population estimates (1990–2030) by 5-years age groups [9]. Mortality was also standardized on the World Health Organization (WHO) estimations for global mortality standardization [10], this allows for a global comparison of mortality rates. Socio-demographic data used to evaluate differences and possible risk factors of CVD were obtained from data sources such as Mexico’s National Institute of Statistics and Geography (INEGI) and Mexico’s National Council on Political and Social Development (CONEVAL) Table 1.

**Table 1:** Socio-demographic variables used for analysis at municipal level

Education Level	[0] “Limited or No Education”
	[1] “From 1 to 6 Years”
	[2] “From 6 to 9 Years”
	[3] “From 9 to 12 Years”
	[4] “More than 12 Years”
Health Insurance Coverage	Percentile distribution groups from 1 to 10.
	1 (No coverage) – 10 (Full coverage)
Municipal Size	[1] Rural: Less than 2,500 population.
	[2] Small Towns: More than 2,500 and less than 10,000.
	[3] Urban: More than 10,000 population and not classified as metropolitan.
	[4] Metropolitan: Municipalities classified by Mexico’s Population Council [11].

Education level and insurance percentage is an average calculated from single death cases for each municipality

A total of 1.7 million deaths due to CVD (ICD-10 I00-I99) were examined for the 1998–2012 period and ecological risk factors data was generated using individual education and insurance data from each death case. The mortality data is a yearly compilation by the Mexican government of all deaths across the country described with individual ICD-10 codes, this data is publicly available and was downloaded from the Mexico’s General Directorate on Health Information (DGIS) [12].

We used the direct method of mortality adjustment to calculate age-adjusted mortality rates [13], providing a comparable measure of mortality to adjust for unequal age distribution. The data is comparable over time and from the highest level of aggregation (national level) to the lowest level of observation (locality). Socio-demographic variables such as education and health insurance coverage were linked to each municipality and year. A geographic grouping variable was also created based on municipal population size. We gathered individual data from each death about education and classified by education level that was later aggregated as an average for the municipality. Similarly insurance was measured using a binary of having any kind of insurance and calculating an percentage of coverage based on total deaths. Underestimation of mortality has been previously described across Mexico [5], this underreporting is expected to be random but no information is available on the percent of misreporting for CVD mortality at the municipal level.

This study was designed as a cross-sectional time series analysis utilizing all mortality cases in Mexico associated with CVD for the years 1998 to 2012. The main outcome for our study was age-adjusted mortality rate for each municipality over time. We selected cases using ICD-10 codes from I00 through I99 in the main cause of death for each death case, generating a specific CVD dataset. The educational component in our study was evaluated using an ordinal variable as shown in Table 1, generated from reported education attainment in each death certificate. Insurance coverage was calculated for each municipality using the percentage of those with health insurance at time of death and later distributed in percentage deciles. Municipal size was classified based on population size as shown in Table 1 and a Metropolitan area classification from the National Population Council. Initial exploratory analyses of the data with histograms were used to check for normal distribution. Factors that showed a significant p-value of more than 0.1 in univariate analysis were considered for multivariate modelling. We used a mixed effect linear model with random effect estimation and repeated measurements to compare and predict the main outcome variable (mortality rate), the covariates (education and insurance) and the geographic delimitations (border/non-border and municipal size). Alpha level of 0.05 was used for all analysis; all analysis were performed using SPSS v22.

## Results

**Fig. 1:**
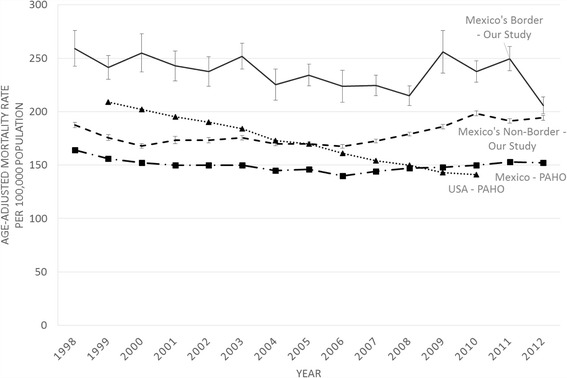
Cardiovascular age-adjusted mortality rates for Mexico’s Border and Non-border municipalities from 1998 to 2012. Comparison to data from the Pan American Health Organization. Mortality standardized to 5 years age groups and represent cardiovascular deaths per 100,000 population

Data from the Pan American Health Organization (PAHO) basic health indicator repository [14] shows that age-adjusted mortality in Mexico due to cardiac and circulatory disease is on average 149 · 8 deaths per 100,000 population. In our study, the average mortality at the Mexican side of the US-Mexico border and non-border region was 230 · 2 and 177 · 8 per 100,000 population, respectively. Modelled results were compared over time using a mixed-effect linear regression model for comparison between similar size municipalities and controlling for ecological confounding variables as shown in Table 1. Figure 1 shows the results of the comparison between the mean age-adjusted mortality rates for border and non-border municipalities in Mexico, and compares to the US and Mexico data from the PAHO.

As can be seen in Fig. 1, there’s a consistently higher mortality rate in the border municipalities, which is supported by other studies that have reported higher prevalence and mortality due to CVD in US border municipalities [15–17]. We can also observe that the non-border region has a growing CVD mortality trend compared to a downward trend in the border region. The non-border region in our study is similar to that of the Mexico-PAHO with slight variation, which can be explained by possible differences in the population used for mortality adjustment. Finally, we can also observe that the CVD mortality in the US is rapidly declining, contrary to the trend from Mexico.

The linear model in Table 2 shows that after adjusting for education level, insurance coverage and municipal size, the border region shows a mean difference in CVD deaths of 78 · 5 (95% CI 58 · 7-98 · 3, *p* < 0 · 001) compared to the non-border region. Additionally, the covariates included in the linear model predict changes in mortality based on the attribute from each municipality in each of the groups within education, insurance or municipal size. There is an estimated reduction in mortality of 12 · 6 (95% CI 11 · 4-13 · 8, *p* < 0 · 001) less CVD deaths per municipality size change (form Rural to Small Town, to Urban and to Metropolitan). We used deciles to measure insurance coverage (0-100%) in all municipalities and observed an increase of 3 · 55 (95% CI 3 · 1-4 · 0, *p* < 0 · 001) more death cases per decile increase (1–10) towards higher insurance coverage. Education level showed a reduction in CVD mortality of 8 · 6 (95% CI 5 · 5-11 · 8, *p* = 0 · 001) less cases per each group change (Limited/No Education, 1–6 years, 6–9 years, 9–12 years and more than 12 years) towards the higher level of education group. In the case of time, our model showed an estimated increase of 0 · 3 (95% CI 0 · 001-0 · 6, *p* = 0 · 050) more deaths per year in the non-border. The Mexican border municipalities showed an estimated decrease of 2 · 9 deaths per year (95% CI 0 · 75-5 · 0 *p* = 0 · 008) compared to non-border municipalities. This is consistent with the down trending cardiovascular mortality seen in Fig. 1 for the border municipalities.

**Table 2:** Mixed-effect linear model of age-adjusted mortality rates due to cardiovascular disease in Mexico from 1998 to 2012 by Border and Non-border regions

	B	Standard Error	*p*-value	95% Confidence Intervals
Intercept (Non-Border)	197 · 7	20 · 43	<0 · 001	113 · 8, 281 · 5
Border	78 · 5	10 · 10	<0 · 001	58 · 7, 98 · 3
Municipal Size [Rural, Small Town, Urban, Metropolitan]	−12 · 6	0 · 61	<0 · 001	−13 · 8, −11 · 4
Education Level [0–1, 1–6, 6–9, 9–12, >12 years]	−8 · 6	1 · 60	<0 · 001	−11 · 8, −5 · 5
Insurance [Deciles 0–100%]	3.6	0 · 24	<0 · 001	3 · 1, 4 · 0
Time [15 years]	0 · 3	0 · 15	0 · 050	−0 · 001, 0 · 60
Border * Time	−2.9	1 · 07	0 · 008	−5 · 0, −0 · 75

## Discussion

We found that after controlling for socio-demographic variables, the Mexican border municipalities have a significantly higher mortality due to CVD compared to the non-border municipalities in Mexico. Although we can observe a downward mortality trend in the border municipalities, the non-border municipalities are increasing its CVD mortality trend. Our linear model estimated the predictive effect of education, insurance and municipal size, observing that higher education and higher municipal size had a lowering effect in CVD mortality while insurance coverage as well as time had an increasing effect in CVD mortality.

To the best of our knowledge, these findings are the first to compare border versus non-border municipalities across Mexico with adjustment of common social and demographic factors. Results from our study are in agreement with findings from those of Morales LS [18] that described how lower levels of education lead to higher CVD risk factors in a sample of Mexican and US Nationals. However, in their study, they only focused on risk factors rather than mortality rates. They also found that risk factors are more common in Mexican Americans born in Mexico and living in the US than Mexican nationals who lived in Mexico. This supports our hypothesis that mortality risk in the border region of Mexico is likely resulting from similar risk factors to those living in the US. These results can help support research towards more specific factors and differentiate regional mortality distribution that can explain the disproportionate effect of some risk factors. Our results are supported by findings of the overall mortality rates reported by the PAHO [14]. Proper treatment and early detection strategies used in the US has led to a consistent and a rapid reduction in CVD mortality as seen in Fig. 1. These strategies can be implemented in the border municipalities or other affected regions of Mexico with the potential of lowering CVD deaths. The border region can provide an initial framework of CVD prevention, expanding to non-border municipalities that are rapidly closing the CVD mortality gap.

The reasons for an increase in mortality from CVD and especially in the border area are expected to be multifactorial. One aspect might be related to body composition and ethnic or genetic susceptibility. It has been shown that regardless of different risk factors or time living in the US, CVD is higher amongst Mexican Americans compared to other ethnic and racial groups in the US [4, 17, 18]. Graham [19] has identified a higher prevalence of risk factors in most minority groups such as Native American, Mexican-American, African American and Asian-American. This in turn leads to risk factors that go unrecognized and untreated, eventually increasing morbidity and mortality. Similarly, it has been reported by Palaniappan [20] and Araneta [21] how normal laboratory values can vary in Asian-Indian, Japanese-American and Pacific-Islander, and could explain some ethnic-specific relationship with CVD in Mexican-Americans. These studies show us that regardless of place of birth, Mexican-American, and other minorities are disproportionately affected by CVD in the US [22].

Population studies are at risk of assuming relationships known as ecological fallacy. We have evaluated CVD mortality in Mexico and have observed that after adjusting for population distribution, mortality is presently higher in the Mexican border municipalities compared to the non-border municipalities of Mexico. But this relationship can be skewed by smaller population pockets that are disproportionately affected by CVD mortality or by a continuous population movement that is characteristic of the US-Mexico border. Even with efforts to adjust for population size and migration among other elements, the effect that complex societal and individual risk factors could lead to erroneous assumptions associated with mortality data and has to be interpreted with caution. We cannot assume a causal relationship of CVD mortality with the selected covariates as individual data has not been evaluated.

We wanted to see if a higher population density and urban setting can partially answer this question since some cities in the border area are more developed than the rest of Mexico. Based on our multivariate analyses, this was not the case and larger cities had lower mortality than smaller cities regardless of border region. This can be due in part of the superior infrastructure, modern equipment, proximity to care and a larger availability of caregivers in metropolitan areas that could be affecting mortality, but these covariates need to be further studied. The percentage of insurance coverage shows a counterintuitive effect that could be explained by the recent implementation of universal healthcare across Mexico, this issue needs further investigation. This effect of insurance and CVD mortality could be important for future studies, as we have noted an opposed effect compared to other studies. Insurance could be confounding our results as insurance is being provided regardless of disease, income or socio demographic characteristics. And this is important as it can tell us that regardless of insurance, other factors are having a larger effect on mortality.

This higher CVD mortality in the border area that we show might be due to effects of the proximity to the US in the US-Mexico border, and could explain how US acculturation [4, 16] as well as cross-border mobility [23] can be leading to higher mortality in the Mexican border municipalities without the equal access to treatment and healthcare as in the US [3]. Or it could be related to other unique factors for the border area Mexican population.

Based on the linear model we were able to estimate the initial effect of available covariates, with these broad ecological variables we cannot assume a clear-cut effect as we weren’t able to include known risk factors associated with CVD mortality at the local level. The lack of CVD risk factor data at the municipal level limits our study in understanding the regional effect of CVD and limits the detection of specific factors to each municipality. Still, these results suggest that the US-Mexico border region is being affected by higher CVD mortality but that the non-border region is transitioning and increasing their mortality trend.

In this study we tested at the municipal level in all of Mexico to increase the power of the results. We used age-adjusted and standardized mortality rates to compare US-Mexico border municipalities to non-border municipalities for the time period 1998–2012, and controlled for available municipal socio-demographic data as an initial step to evaluate risk factors in CVD mortality. A strength of our study was our ability to compare all of Mexico’s cities based on density of population, education, health insurance and whether it is a border or non-border area. Other studies evaluating mortality and CVD risk factors have been carried out with national and state estimates or have been done in isolated populations and/or controlled environment [18], attenuating the effect of certain risk factors that can be inducing a higher prevalence of CVD disease and increasing deaths. Although our hypothesis was supported statistically, the risk factors that have been described in more detail by other studies (smoking, obesity, HDL cholesterol and LDL cholesterol among others) couldn’t be included as data was not available at the municipal level. Further work should therefore include CVD-specific risk factors with common socio-demographic variables of each municipality. And further work need to be done on the US side of the border with comparison to their Mexican counterparts. We are aware of the effect missing data can have on health and mortality assumptions with large ecological studies. An advantage of this study is that data gathered has been noted to have random missing data and we have further adjusted for the random effect of each municipality. Although we expect more missing data in rural municipalities, we have compared between equal size municipalities that would similarly have missing data and have a better predictive effect of cardiovascular mortality.

This study indicates that the US-Mexico border municipalities have higher CVD mortality that could be explained by region-specific risk factors. Proximity to the US-Mexico border can explain some of the difference of CVD mortality as compared to the non-border municipalities.

Our results provide compelling evidence to further evaluate the effects of the US-Mexico border and its dynamic region on CVD mortality and other chronic diseases, as it provides the unique combination of developed and developing countries with similar risk factors and different health policies. Suggesting that some of the preventive strategies applied in the US can have an effect in the Mexican border municipalities and the rest of Mexico.

## Appendix

**Table 3:** Age-Adjusted Mortality Rate (WHO Methodology) by Year and Region

		Border and Non-Border				Border				Non-Border					
		n	Mean	Std. Dev.	Var.	n	Mean	Std. Dev.	Var.	n	Mean	Std. Dev.	Var.	Rate Ratio	Sig.
Year	1998	2308	188.61	117.45	13795.64	38	259.15	102.13	10431.46	2270	187.43	117.35	13771.84	1.38	<0.001
	1999	2318	176.79	119.66	14319.52	37	241.47	67.80	4596.90	2281	175.74	120.04	14410.33	1.37	<0.001
	2000	2339	169.10	101.55	10313.36	37	254.91	108.50	11771.62	2302	167.72	100.87	10174.72	1.52	<0.001
	2001	2316	174.53	156.93	24626.86	37	242.88	85.04	7231.48	2279	173.42	157.59	24835.47	1.40	0.008
	2002	2346	174.29	118.16	13960.91	37	237.48	84.69	7172.22	2309	173.28	118.35	14007.80	1.37	0.001
	2003	2351	176.75	103.97	10810.76	37	251.84	73.72	5433.97	2314	175.55	103.96	10807.48	1.43	<0.001
	2004	2357	170.79	94.39	8909.16	38	225.17	89.57	8022.91	2319	169.90	94.22	8877.89	1.33	<0.001
	2005	2365	170.71	89.65	8036.26	38	234.24	62.78	3941.37	2327	169.67	89.65	8037.83	1.38	<0.001
	2006	2378	168.33	87.58	7670.45	37	223.86	91.11	8301.22	2341	167.45	87.26	7614.49	1.34	<0.001
	2007	2368	173.26	82.51	6807.28	38	224.56	59.26	3511.84	2330	172.43	82.58	6818.93	1.30	<0.001
	2008	2379	179.69	85.99	7393.45	38	214.83	57.09	3258.72	2341	179.12	86.26	7441.61	1.20	0.011
	2009	2393	187.05	104.44	10907.81	37	255.92	120.52	14524.28	2356	185.97	103.83	10781.48	1.38	<0.001
	2010	2398	198.97	114.82	13183.59	37	237.46	61.31	3758.44	2361	198.36	115.37	13309.35	1.20	0.040
	2011	2388	192.21	101.15	10231.00	37	249.58	69.21	4790.20	2351	191.30	101.32	10266.07	1.30	0.001
	2012	2398	194.50	109.35	11956.65	38	205.83	48.46	2348.84	2360	194.32	110.05	12110.31	1.06	0.520
	Average		179.76	107.65	11589.50		237.17	81.64	6664.62		178.83	107.77	11615.06		

*Std. Dev.* Standard Deviation, *Var.* Variance, *Sig.* Significance by *t*-test between border/non-border

**Table 4:** Age-Adjusted Mortality Rate (WHO Methodology) by Year and Population Size

		Rural				Mixed-Urban				Urban				Metropolitan			
		n	Mean	Std Dev	Var.	n	Mean	Std Dev	Var.	n	Mean	Std Dev	Var.	n	Mean	Std Dev	Var.
Year	1998	306	220.11	225.60	50893.32	886	189.30	114.56	13124.79	753	177.99	62.85	3949.54	363	182.41	60.29	3634.44
	1999	305	210.74	257.96	66541.03	894	173.71	97.83	9571.29	754	167.04	57.17	3268.85	365	176.10	64.40	4146.73
	2000	322	203.98	178.96	32027.47	896	166.21	102.42	10490.63	755	158.32	59.25	3510.24	366	167.72	59.87	3584.47
	2001	303	235.25	356.76	127278.73	893	168.91	119.11	14186.57	755	157.84	57.86	3347.97	365	172.39	74.81	5596.08
	2002	320	225.08	232.15	53892.96	902	165.62	111.28	12382.54	758	164.87	57.46	3301.66	366	170.76	50.35	2535.45
	2003	324	213.31	183.88	33812.39	904	172.88	105.81	11195.39	758	166.75	57.07	3256.65	365	174.62	61.69	3805.45
	2004	322	201.88	162.47	26396.39	911	168.72	99.89	9977.85	760	161.14	52.52	2758.44	364	168.66	51.31	2632.50
	2005	326	179.13	146.79	21548.66	912	169.99	96.39	9291.59	761	169.51	57.73	3333.27	366	167.49	51.50	2652.16
	2006	336	185.62	141.66	20067.06	916	168.81	96.59	9329.25	760	162.30	52.82	2789.95	366	163.77	44.43	1973.81
	2007	330	196.06	137.33	18860.32	911	170.73	85.99	7394.53	760	169.80	52.80	2787.78	367	166.23	48.42	2344.82
	2008	331	201.01	147.80	21845.93	920	178.48	87.00	7568.40	761	176.89	55.63	3095.02	367	169.31	48.69	2370.38
	2009	345	220.76	186.49	34778.75	920	185.52	105.50	11130.27	761	181.58	56.96	3244.79	367	170.58	50.03	2503.48
	2010	351	238.32	213.98	45789.16	919	197.97	109.50	11989.31	761	194.14	60.46	3655.70	367	173.84	52.17	2721.88
	2011	338	229.83	182.68	33372.23	922	192.14	98.79	9760.20	761	187.02	58.29	3397.79	367	168.48	49.53	2453.58
	2012	348	229.44	188.04	35360.08	922	198.89	115.10	13248.71	761	186.81	57.99	3362.39	367	166.30	48.28	2330.62
	Average		212.77	202.50	41008.20		177.92	104.01	10818.30		172.15	58.19	3386.24		170.57	55.05	3030.43

*SE* Standard Error, *95% CI* 95 Percent Confidence Interval, *Sig.* Significance

## Abbreviations

CONAPO: Mexico’s National Population Council
CONEVAL: Mexico’s National Council on Political and Social Development
CVD: Cardiovascular Disease
DGIS: Mexico’s General Directorate on Health Information
ICD-10: 10^th^ International Classification of Disease
INEGI: Mexico’s National Institute of Statistics and Geography
PAHO: Pan American Health Organization
RFs: Risk Factors
US: United States
WHO: World Health Organization
